# *In Vitro* Inhibitory Mechanism of Polyphenol Extracts from Multi-Frequency Power Ultrasound-Pretreated Rose Flower Against α-Glucosidase

**DOI:** 10.3390/foods13213421

**Published:** 2024-10-27

**Authors:** Chao Zhang, Ming Feng, Bimal Chitrakar, Fan Yang, Benxi Wei, Bo Wang, Cunshan Zhou, Haile Ma, Xianli Gao, Baoguo Xu

**Affiliations:** 1 School of Food and Biological Engineering, Jiangsu University, Zhenjiang 212013, China; chaozhang202410@163.com (C.Z.); fengmin0616@163.com (M.F.); bxwei@ujs.edu.cn (B.W.); wangbo670@163.com (B.W.); mhl@ujs.edu.cn (H.M.); gaoxianli@ujs.edu.cn (X.G.); 2 College of Food Science and Technology, Hebei Agricultural University, Baoding 071001, China; bimalchitrakar@gmail.com; 3 College of Food Science and Engineering, Nanjing University of Finance and Economics, Nanjing 210023, China; 4 Institute of Food Physical Processing, Jiangsu University, Zhenjiang 212013, China

**Keywords:** polyphenols, α-glucosidase, inhibitory mechanism, spectroscopy, molecular docking

## Abstract

This paper explored the in vitro inhibitory mechanism of polyphenol-rich rose extracts (REs) from an edible rose flower against α-glucosidase using multispectral and molecular docking techniques. Results showed that REs had an inhibitory effect on α-Glu activity (IC_50_ of 1.96 μg/mL); specifically, the samples pretreated by tri-frequency ultrasound (20/40/60 kHz) exhibited a significantly (*p* < 0.05) stronger inhibitory effect on α-Glu activity with an IC_50_ of 1.33 μg/mL. The Lineweaver–Burk assay indicated that REs were mixed-type inhibitors and could statically quench the endogenous fluorescence of α-Glu. REs increased the chance of polypeptide chain misfolding by altering the microenvironment around tryptophan and tyrosine residues and disrupting the natural conformation of the enzyme. Molecular docking results showed that polyhydroxy phenolics had a high fit to the active site of α-Glu, so REs with high polymerization and numerous phenolic hydroxyl groups had a stronger inhibitory effect. Therefore, this study provides new insights into polyphenol-rich REs as potential α-glucosidase inhibitors.

## 1. Introduction

Diabetes mellitus (DM) is a metabolic disorder characterized by hyperglycemia, caused by abnormalities in fat, protein, and carbohydrate metabolism due to insulin resistance or insulin deficiency [1]. Globally, about 537 million people aged 20–79 years have diabetes, and 643 million are expected to have diabetes by 2030 (11.3% of the total population); by 2045, this number is predicted to jump to 783 million (12.2%) [2]. Diabetes mellitus is divided into four main types, among which type II diabetes mellitus is the most prevalent [3], often accompanied by hyperglycemia, obesity, cardiovascular disease and/or other diseases. Its root cause lies in the digestion and absorption of some starchy foods [4], so diabetic patients need lifelong dietary control and medication. Alpha-glucosidase (α-Glu) hydrolyzes carbohydrates into glucose, which is then absorbed into the blood, resulting in elevated blood sugar and causing damage to the human body, so controlling the activity of carbohydrate-digesting enzymes is considered to be an effective way to lower blood glucose [5,6]. However, many hypoglycemic drugs (for example, acarbose, voglibose, etc.) can be used for a short period of time to control the activity of α-glucosidase, but a long-term administration of such drugs may cause toxic side effects in the body, such as liver and gallbladder lesions, diarrhea, nausea, vomiting, lactic acidosis, hypoglycemia, weight gain, skin reactions, and gastrointestinal disorders, resulting in a heavy financial burden to patients [7].

Natural polyphenolic compounds contained in plants have been found to be safer natural alternatives compared to hypoglycemic drugs [8]. Polyphenols including populin, quercetin, and tannins have been previously reported to have inhibitory activity against carbohydrate-digesting enzymes [9]. Multiple phenolic compounds are usually present in plants at the same time, which are of greater interest in their involvement in blood glucose regulation due to the interactions among the different components [10].

As one of the traditional food and medicine resources in China, the rose is rich in nutrients such as polyphenols, polysaccharides, proteins, organic acids, minerals, and vitamins [11,12]. A daily intake of 40 g of rose hip powder over a period of 6 weeks has been shown to substantially decrease cardiovascular risk in obese individuals, through the reduction in systolic blood pressure and the lowering of plasma cholesterol levels [13]. In our previous study, we found that the polyphenol content accumulated by ultrasonically treated roses increased and identified a total of 19 phenolic compounds from rose extracts, with higher levels of catechin, quercetin-3β-D-glucoside, phloridzin, proanthocyanidin B_2_, gallic acid, and rutin [14]. The ethanolic extract of rose, which was fractionated by sequential solvent extraction, was found to have a significant hypoglycemic effect on diabetic rats; the extract was found to have significant antidiabetic activity (50–62%) in diabetic rats [15]. Thus, roses exhibit significant advantages in terms of antioxidant, anti-inflammatory, anticancer, antibacterial, and antidiabetic properties due to the presence of polyphenols [16], which produce various beneficial effects on human health, providing significant potential in healthcare.

The inhibitory effect of natural products on starch-digesting enzymes is related to structural features such as the types of basic structural units, the degree of polymerization, and the types of linkage bonds of their constituents [17]. Compared to other polyphenols, proanthocyanidins have a greater affinity for carbohydrate enzymes, which is attributed to their numerous hydroxyl groups and complex structure [18]. From our previous metabolomic studies, we found that the phenolic compounds in REs were polyhydroxy compounds and rose polyphenols were highly polymerized with numerous hydroxyl groups compared with other monomeric polyphenols. However, the type of inhibition of α-Glu by polyphenol-rich REs and further inhibitory mechanisms have not been elucidated.

Enzyme kinetics is the scientific study of the ability of enzymes to bind substrates for a catalytic reaction, and obtains data on the reaction rate between the reacting substrate and the enzyme [19]. The primary methods for studying molecular interactions and mechanisms of action are fluorescence spectroscopy, circular dichroism, and other spectroscopic methods. Molecular docking can help us visualize and understand the binding mode between the substrate and enzyme in a more intuitive and in-depth way [20]. Therefore, this work evaluated the type of in vitro inhibitory effect of polyphenol-rich REs on α-Glu activity through enzyme kinetic methods and analyzed the binding of REs to α-Glu by multispectral methods and molecular docking means, aiming to understand the mechanism of interaction between polyphenol-rich REs and α-Glu.

## 2. Materials and Methods

### 2.1. Raw Materials

**Figure 1:**
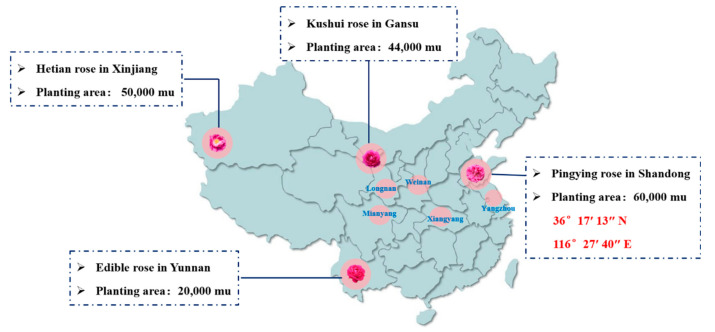
The main rose planting areas and rose varieties in China.

Fresh roses (Pingying roses) shown in Figure 1 with uniform size and no mechanical damage were selected for the experiment, which were transported to the laboratory and kept under refrigeration (4 °C). α-Glu was obtained from yeast and purchased from Source Leaf Bio-technology Co., Ltd. (Shanghai, China), while all other reagents were purchased from Sinopharm Chemical Reagent Co. (Shanghai, China).

### 2.2. Preparation of Polyphenol-Rich Rose Extracts

The fresh edible rose flowers were ultrasonicated under a tri-frequency of 20/40/60 kHz with a power of 150 W for 5 min using multi-frequency power ultrasound equipment self-designed by Jiangsu University (MFPU-1, Zhenjiang, China). Afterward, the untreated and ultrasonicated rose flowers were lyophilized in a freeze-dryer (Epsilon 2-6D LSCplus, Martin Christ Ltd., Osterode am Harz, Germany). The freeze-dried rose petals were crushed by a pulverizer. An accurately weighed rose powder was extracted with 50% ethanol solution at a material–liquid ratio of 1:45 g/mL by using ultrasonic-assisted extraction (an ultrasonic power of 300 W and a dual frequency of 20 + 28 kHz in the simultaneous working mode) for 30 min according to our previous study [21]. The solution was then centrifuged at 11,000× *g* for 10 min and the supernatant was lyophilized. The powder obtained was used for subsequent experiments. The extracts from the roses were dissolved in dimethyl sulfoxide to prepare RE solutions.

### 2.3. Analytical Methods

#### 2.3.1. Determination of Total Phenol Content

The total phenolic content (TPC) was based on our previous study [21]. The TPC of the rose extract was determined by the Folin–Ciocalteu method using gallic acid as a standard.

#### 2.3.2. Determination of α-Glucosidase Activity

In vitro hypoglycemic activity was expressed as the effect of the inhibition of α-glucosidase (α-Glu) activity, and such activity was determined according to the method of Yang et al. [22] with slight modifications. Briefly, 20 μL of α-Glu (1 mg/mL) was mixed with 20 μL of RE solution with different concentration gradients, and 90 μL of a PBS (0.1 mol/L, pH 6.8) buffer was added to a 96-well plate, and 20 μL of the reaction substrate 4-nitrophenyl-α-D-galactoside pyranopyranoside (pNPG) solution (1 mmol/L) was added immediately. The reaction was stopped by adding 50 μL Na_2_CO_3_ solution (0.2 mol/L) after 20 min of the reaction at 37 °C, and the absorbance value was measured at 405 nm after cooling to room temperature.

#### 2.3.3. Determination of the Type of α-Glucosidase Inhibition

The method described by Di et al. [23] was used for the determination of α-Glu inhibition. The type of inhibition of α-Glu by REs was judged according to the Lineweaver–Burk double inverse curve.

#### 2.3.4. Fluorescence Spectrometry

The fluorescence spectra were determined according to the method of Wang et al. [24] using a spectrometer (Cary Eclipse, Varian Medical Systems, Palo Alto, CA, USA). The α-Glu was mixed with different concentrations of REs (final concentrations of 0, 0.26, 0.52, 1.04, 2.08, and 4.17 μg/mL) and incubated for 5 min at 298, 304, and 310 K, respectively. The fluorescence spectra of the samples were then determined by transferring the samples into quartz cuvettes (1 cm) at an excitation wavelength of 280 nm and an emission wavelength ranging from 300 to 400 nm, while the slit width was 5.0 nm.

Based on the research of fluorescence quenching, the thermodynamic parameters, namely *ΔH°* (enthalpy change), *ΔS°* (entropy change), and *ΔG°* (free energy change), were calculated by using thermodynamic equations.

(1) $$\log\left(\frac{F0-F}{F}\right)=logKa+nlog[Q]$$

(2) $$lnKa=-\frac{\Delta H{}^{\circ}}{RT}+\frac{\Delta S{}^{\circ}}{R}$$

(3) ΔG°=ΔH°−TΔS°

Synchronized fluorescence spectroscopy was determined using the intervals between excitation and emission wavelengths (Δλ) as 15 nm and 60 nm, respectively.

Three-dimensional fluorescence spectrometry was analyzed using an emission wavelength of 200~500 nm and an excitation wavelength of 200~500 nm.

#### 2.3.5. Circular Dichroic Scanning

The α-Glu was mixed with REs and incubated for 5 min at 25 °C, and the wavelength range of the circular dichroism (CD) scan was set to 200~250 nm. The background was corrected with a PBS buffer. The secondary structure content of α-Glu was further calculated using CD pro software https://sites.google.com/view/sreerama (Narasimha Sreerama Research Group, Fort Collins, CO, USA) [25].

#### 2.3.6. Molecular Docking

Molecular docking simulations were performed according to the method of Wang et al. [26] with some modifications. The website (http://cadd.labshare.cn/cb-dock2/) “URL (accessed on 29 July 2022)” was used for molecular docking simulation tests [27]. The 3D structures of acarbose, catechin, quercetin-3β-D-glucoside, proanthocyanidin B_2_, gallic acid, and rutin were downloaded from the PubChem website (https://pubchem.ncbi.nlm.nih.gov/ (accessed on 29 July 2022)). As noted in earlier research [28], the crystal structure of α-glucosidase has not been widely reported; its homologous structure isomaltose from *Saccharomyces cerevisiae* was utilized to perform the docking assay from the https://www.ncbi.nlm.nih.gov/ (accessed on 29 July 2022) website to download the α-Glu crystal structure (PDB ID: 3A4A). The amorphous dehydrogenation of α-Glu and the addition of missing hydrogen atoms was performed. After docking was completed, the molecular docking visualization images were exported using Discovery Studio Client. The interfacial amino acid residues on α-Glu can be derived from the docking results.

### 2.4. Statistical Analysis

The results of the trials were statistically analyzed using SPSS 25.0 statistical software using Duncan to compare the one-way variance between groups, with at least three repetitions of each group, while differences in the means at *p* < 0.05 level were considered significant.

## 3. Results and Discussion

### 3.1. Analysis of the Inhibitory Activity of Polyphenol-Rich Rose Extract on α-Glucosidase

The inhibitory effect of REs from the fresh rose samples pretreated by tri-frequency ultrasound (20/40/60 kHz) on α-glucosidase activity was compared using untreated REs as the control. As can be seen from Table 1, the inhibitory effect of REs on α-Glu activity was significantly (*p* < 0.05) increased in the ultrasound-treated group with an IC_50_ of 1.33 μg/mL. Ultrasound as a non-thermal technology has been widely used in food processing, such as extraction [21], freezing [29], drying [30], sterilization [31], etc. In our previous study, it was found that when plants were exposed to external stress conditions, the cells suffered oxidative damage, which led to a series of defense responses, such as protecting themselves from damage by producing secondary metabolites with antioxidant activity. Therefore, when the fresh rose was treated with ultrasound, rose cells enhanced their tolerance to ultrasound stress by accumulating phenolics. Thus, ultrasonic stress significantly (*p* < 0.05) promoted the accumulation of rose polyphenols (shown in Table 1), which further enhanced the inhibitory effect of the extract on α-Glu activity. Quispe-Fuentes et al. [32] reported that extracts of freeze-dried samples with a higher retention of phenolic compounds had a stronger inhibitory effect on α-Glu compared to samples of Maki tree fruits obtained by other drying methods. Chen et al. [33] found similar results with polyphenol-rich extracts of *Lycium barbarum* fruit, which had a significant inhibitory effect on α-Glu, 5–150 times stronger than that of acarbose. Therefore, it is necessary to elucidate the constitutive relationship in further studies to fully understand the mechanism of α-Glu inhibition by polyphenol-rich REs.

### 3.2. Determination of the Type of α-Glucosidase Inhibition by Rose Extracts

**Figure 2:**
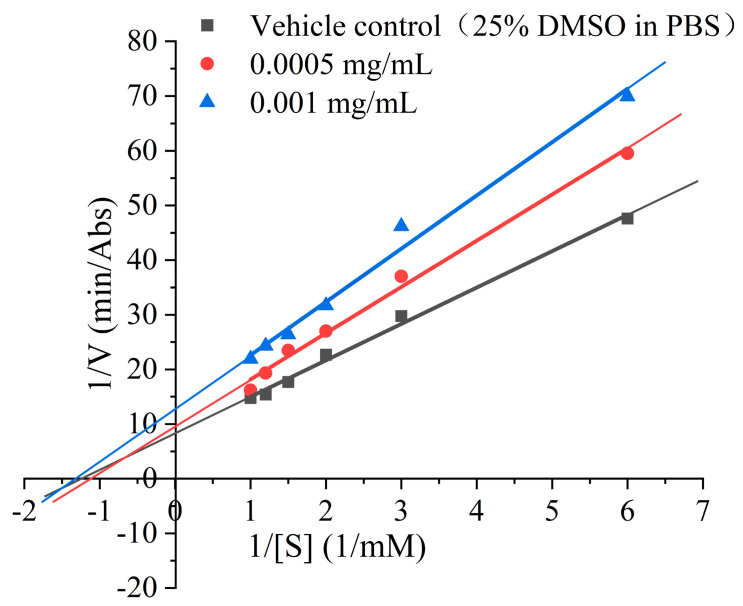
Effect of rose extracts (REs) on α-Glu Lineweaver–Burk curve.

The inhibition mode of REs on α-Glu was identified by utilizing the Lineweaver–Burk plot. As shown in Figure 2, all lines intersected at a point beyond the x- and y-axes in the presence of different concentrations of REs, suggesting that REs were mixed-type inhibitors of α-Glu [34]. Wang et al. [1] found similar results when investigating the kinetics of the inhibition of pancreatic α-amylase by flavonoids from the Lotus leaf. Phenolic compounds have been reported to act as metastable inhibitors that bind to metastable residues around α-Glu and affect enzyme activity by altering the natural conformation and microenvironmental polarity of the enzyme [9,35]. Therefore, further investigation of the effects of REs on the structure of α-Glu would be beneficial in elucidating the possible mechanism of inhibition.

### 3.3. Effect of Polyphenol-Rich Rose Extract on the Fluorescence Spectra of α-Glucosidase

#### 3.3.1. Endogenous Fluorescence Spectroscopy

**Figure 3:**
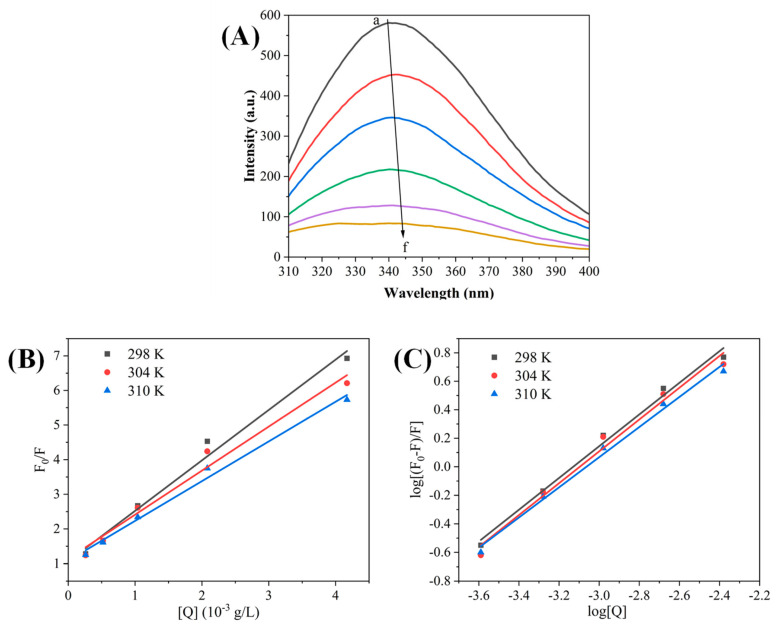
Effect of REs on fluorescence spectra (**A**), Stern–Volmer curves (**B**), and double logarithmic curves (**C**) of α-Glu. (Note: curves a → f correspond to rose extract concentrations of 0, 0.26, 0.52, 1.04, 2.08, and 4.17 μg/mL, and temperatures of 298K).

Protein fluorescence originates from specific amino acid residues including tyrosine, tryptophan, and phenylalanine. Alterations in the microenvironment surrounding these residues can influence enzymatic activity [36]. Therefore, in this study, the fluorescence spectra and intensity changes were analyzed to predict conformational changes upon enzyme–polyphenol interaction. The impact of REs on the fluorescence spectrum of α-Glu is illustrated in Figure 3A, where α-Glu has a strong fluorescence emission peak near 340 nm. Since the maximum excitation wavelengths of tryptophan (Trp) and tyrosine (Tyr) are about 350 nm and 305 nm, respectively, at an excitation wavelength of 280 nm, Trp and Tyr in α-Glu are considered to be the main amino acids responsible for fluorescence emission. As shown in Figure 3A, the fluorescence intensity of α-Glu decreased gradually with increasing concentrations of REs (a to f: 0, 0.26, 0.52, 1.04, 2.08, and 4.17 μg/mL, respectively), indicating that REs can bind to α-Glu and quench its intrinsic fluorescence [24,35]. Meanwhile, this emission peak was gradually redshifted from 340 nm to 345 nm with the increase in RE concentration. The fluorescence quenching and redshift induced by REs substantiated the interaction of REs with α-Glu, and the REs altered the hydrophobic microenvironment of amino acids in the enzyme, particularly in the vicinity of Trp and Tyr residues.

To further investigate the fluorescence quenching mechanism of REs on α-Glu, the fluorescence data were analyzed using the Stern–Volmer equation. The fluorescence quenching of α-Glu was categorized into two forms: static quenching and dynamic quenching. Static quenching involves the formation of non-fluorescent complexes, whereas dynamic quenching results from energy transfer via collisions [24]. Analysis of Figure 3B showed that the Stern–Volmer curves of α-Glu had a good linear relationship at different temperatures (298, 304, and 310 K), and the quenching constants represented by the slopes decreased with increasing temperature (Table 2). These findings indicated that REs can form non-fluorescent complexes with α-Glu and the quenching mode between them is a static quenching mechanism.

In Figure 3C, the Stern–Volmer curve is transformed into a double logarithmic curve using the Scatchard equation, which further obtained the binding constants (Ka) and the number of binding sites (n). Notably, the number of binding sites was close to one at different temperatures, implying the presence of a major binding site for REs on α-Glu. In addition, the higher the temperature, the smaller the Ka value, suggesting that the temperature increase was unfavorable to the stability of the REs and α-Glu complexes, further illustrating the static quenching mechanism.

The thermodynamic analysis was used to further determine the types of interaction forces between REs and α-Glu. Hydrogen bonding, hydrophobic interactions, electrostatic forces, and Van der Waals forces are the intermolecular forces that drive enzyme–ligand binding under normal conditions [24]. Table 2 shows that ΔH°, ΔG°, and ΔS° in the binding reaction of REs with α-Glu were all negative, indicating that the reaction proceeded spontaneously. And it is hypothesized that hydrogen bonding is the main driving force for the interaction of REs with α-Glu [37]. In addition, similar results were reported in studies of α-Glu binding to sesquiterpenes [38].

#### 3.3.2. Synchronized Fluorescence Spectroscopy

**Figure 4:**
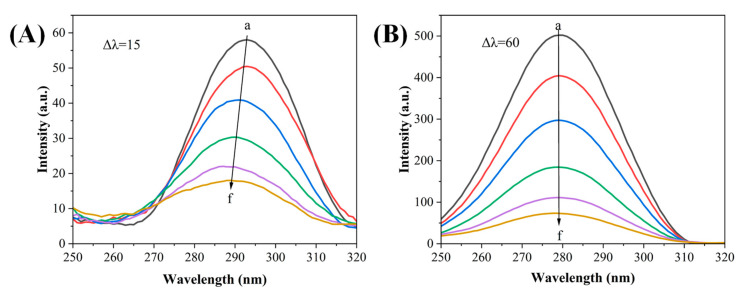
Effect of REs on synchronous fluorescence spectra of α-Glu at Δλ = 15 nm (**A**) and Δλ = 60 nm (**B**) (the curves a → f correspond to rose extract concentrations of 0, 0.26, 0.52, 1.04, 2.08, and 4.17 μg/mL, respectively).

The fluorescence spectra obtained could be used to reflect the microenvironmental changes in Tyr and Trp residues in α-Glu under the excitation and emission wavelengths of 15 nm and 60 nm, respectively. As shown in Figure 4, REs caused fluorescence quenching, which was observed in α-Glu under both synchronized fluorescence conditions. Moreover, REs caused the maximum emission peak wavelength of Tyr residues in α-Glu, which was blue shifted from 293 nm to 289 nm when Δλ was 15 nm (Figure 4A); however, no significant change could be observed when Δλ was 60 nm (Figure 4B). This indicated that REs could bind to Tyr and Trp residues, especially increasing the hydrophobicity of Tyr residues. Similar results were reported for the effect of proanthocyanidin on α-amylase from poplar leaves [24]. The alteration of the polarity of the microenvironment containing amino acid residues was found to increase the chances of the misfolding of the polypeptide chain, which in turn changed the natural conformational structure of the enzyme molecule [25], resulting in the reduction in α-Glu activity by REs.

#### 3.3.3. Three-Dimensional Fluorescence Spectroscopy

**Figure 5:**
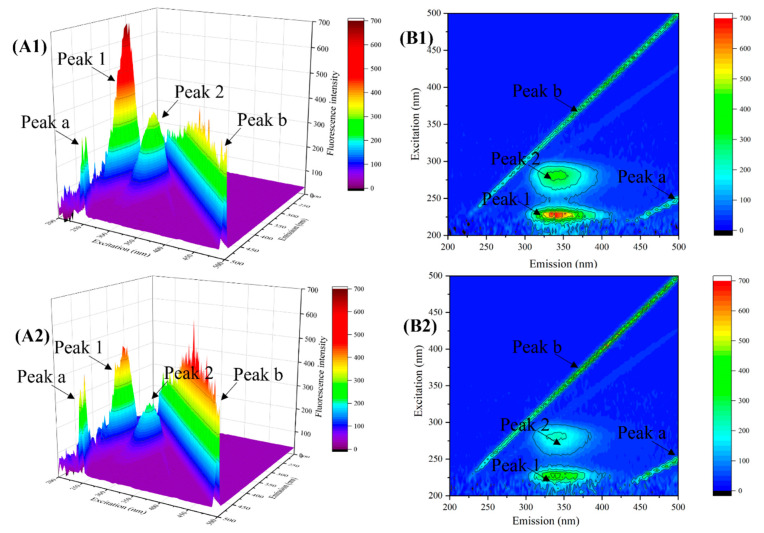
Three-dimensional fluorescence spectra of natural α-Glu (**A_1_**,**B_1_**) and α-Glu–rose extract complex (**A_2_**,**B_2_**).

Three-dimensional fluorescence spectroscopy can be used to analyze changes in the structural properties of amino acids and polypeptide chains in enzyme molecules upon interaction with ligands [39]. As shown in Figure 5(A1,B1), in the 3D fluorescence spectrum of natural α-Glu, there were four obvious main peaks; peaks b and a were the primary and secondary Rayleigh scattering peaks, respectively, which were related to the degree of aggregation of the molecules in solution [40]. Peak 1 mainly reflected the fluorescence characteristics of the carbon–oxygen double bond of the backbone structure of the polypeptide chain, which were related to n-π* leaps, and peak 2 mainly reflected the fluorescence characteristics of the Trp and Tyr residues, which were related to π-π* leaps [41].

As shown in Figure 5(A2,B2), the fluorescence intensity of peaks a and b increased after the addition of REs, which could be attributed to the fact that REs bound to α-Glu to form a new complex, which induced the formation of larger sized particles, leading to a significant increase in the Rayleigh scattering signal. In addition, the fluorescence intensity of peaks 1 and 2 decreased dramatically, indicating that REs interfere with the microenvironmental homeostasis in which the enzyme molecules reside [24]. Specifically, REs may bind in the hydrophobic cavity of the enzyme molecule and alter the hydrophobicity of amino acid residues, leading to changes in the α-Glu polypeptide backbone and thus inhibiting α-Glu activity. In particular, the aromatic ring of polyphenols in REs may form π-π stacking between the aromatic ring of polyphenols and the benzene ring of Try or Tyr residues, inducing the binding of REs to α-Glu in the hydrophobic cavity [42].

#### 3.3.4. Effect of Polyphenol-Rich Rose Extract on the Circular Dichroism of α-Glucosidase

**Figure 6:**
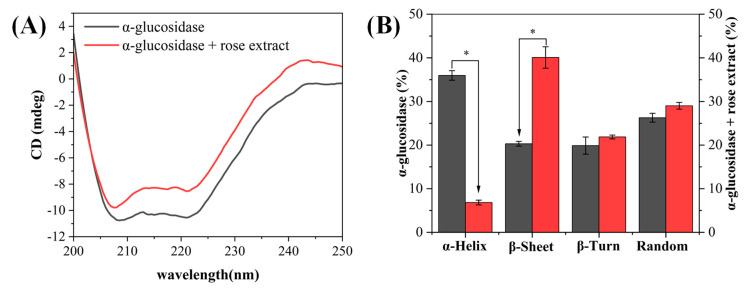
Effect of REs on circular dichroism (**A**) and secondary structure (**B**) of α-Glu. “*” indicates a significant difference between α-glucosidase and α-glucosidase + rose extract.

Circular dichroism (CD) spectroscopy was employed to investigate the influence of REs on the secondary structure of α-Glu. As shown in Figure 6A, two major negative peaks were observed in the CD plot of α-Glu, which were related to the n→π* and π→π* leaps in the α-helix structure of α-Glu [43]. The absorption peak of CD decreased upon the addition of REs, indicating that the interaction of REs with α-Glu resulted in the alteration of the enzyme secondary structure. The CD data were further analyzed to quantify the proportions of the secondary structure components in α-Glu. As shown in Figure 6B, the α-helical content of α-Glu was significantly decreased and the β-sheet content was significantly increased when REs were added. It indicated that the structure of α-Glu exposed to REs became loose and unstable compared to the natural state. These results indicated the existence of an interaction between REs and α-Glu, supporting the findings obtained in fluorescence spectroscopy measurements. Therefore, it can be concluded that the binding of REs to α-Glu increases the chance of polypeptide chain misfolding in the enzyme molecule, which is detrimental to the formation of the α-Glu active center, which in turn leads to a decrease in enzyme activity [44].

#### 3.3.5. Molecular Docking

**Figure 7:**
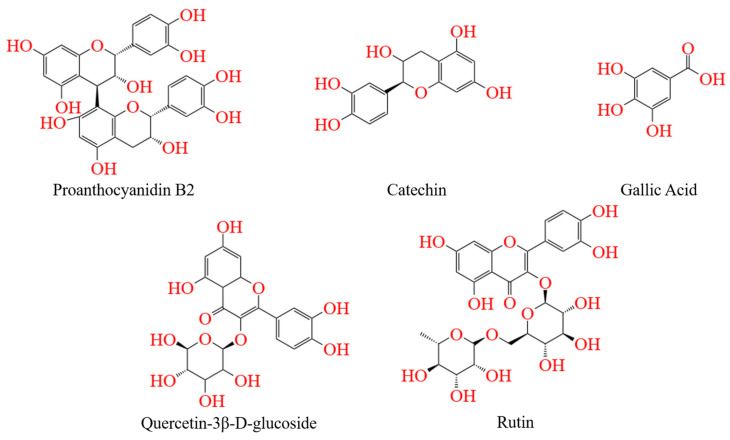
Chemical structures of the potential α-glucosidase inhibitors from REs.

**Figure 8:**
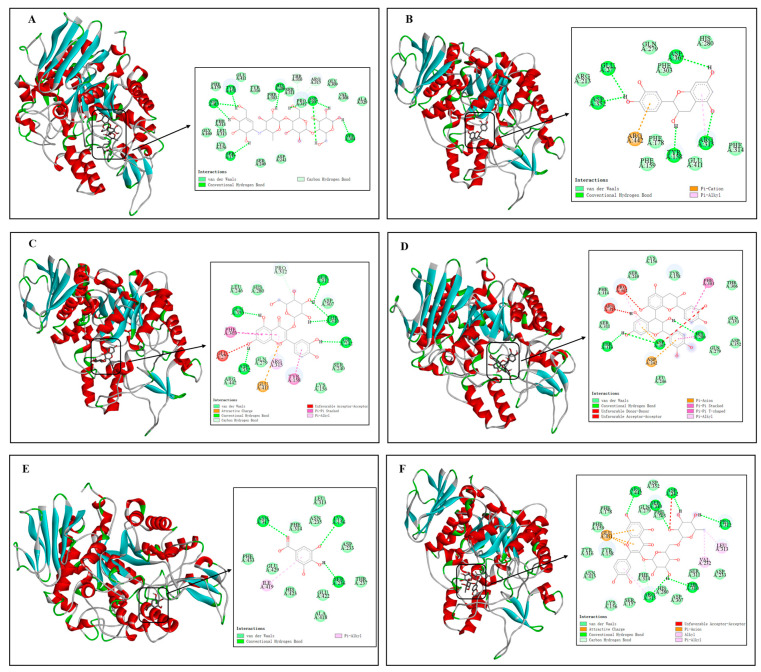
Molecular docking models for the binding of acarbose (**A**) and the major phenolic compounds in roses to α-Glu. All insets in (**A**–**F**) show the amino acid residues bound to the ligands. (**A**)—acarbose; (**B**)—catechin; (**C**)—quercetin-3β-D-glucoside; (**D**)—proanthocyanidin B_2_ (**D**); (**E**)—gallic acid; and (**F**)—rutin.

In order to further predict the binding modes between REs and α-Glu, the major phenolics in the rose (Figure 7) were selected as representatives for molecular docking simulations in this study. Figure 8A–F show the binding details of acarbose, catechin, quercetin-3β-D-glucoside, proanthocyanidin B_2_, gallic acid, and rutin with α-Glu, respectively. It was found that the major phenolics in acarbose and roses were able to bind in the hydrophobic pocket of α-Glu. The predicted binding energies of acarbose, catechin, quercetin-3β-D-glucoside, procyanidin B_2_, gallic acid, and rutin to α-Glu were −8.70, −8.60, −9.60, −9.90, −6.50, and −10.10 kcal/mol (Table 3), indicating a good affinity between polyphenol-rich REs and α-Glu.

By examining the binding details of the inhibitors to α-Glu, it can be found that acarbose, catechin, quercetin-3β-D-glucoside, proanthocyanidin B_2_, gallic acid, and rutin can bind to the amino acid residues in α-Glu through hydrogen bonding. Specifically, acarbose bound via hydrogen bonding to six of its surrounding amino acid residues, including Asn-415, Tyr-158, His-280, Asp-307, Ser-157, and Ser-304, at distances ranging from 3.99 to 5.03 Å. Similarly, catechin bound via hydrogen bonding to five of its surrounding amino acid residues, including Glu-277 (5.89 Å), Asp-352 (4.52 Å), Asp-307 (4.74 Å), Tyr-158 (4.13 Å), and Arg-315 (4.76 Å); quercetin-3β-D-glucoside formed six hydrogen bonds with amino acid residues Gln-353, Ser-311, Thr-310, Asp-352, and Asp-242 at distances of 3.93–4.67 Å. In addition, procyanidin B_2_ formed a Pi-A1ky1 hydrophobic interaction with an amino acid residue, namely Pro-312, at distance of 5.44 Å.

Furthermore, comparing the molecular docking of major phenolic compounds with α-Glu in acarbose and REs, phenolic compounds with high molecular weights had polyhydroxyl groups and complex aromatic ring structures, which favored the enhancement of hydrogen bonding and hydrophobic interactions with α-Glu, leading to a significant decrease in enzyme activity. As a result, procyanidin B_2_ and rutin bound more stably to α-Glu. In summary, potential inhibitors bound to α-Glu through key forces such as hydrogen bonding and hydrophobic interactions, thereby affecting the catalytic activity of key amino acid residues. Since REs are rich in phenolic compounds with polyhydroxyl groups and complex aromatic rings, they were more involved in the binding portion compared to other inhibitors, which may explain the stronger inhibitory effect of polyphenol-rich REs on the activity of α-Glu [2].

## 4. Conclusions

In this study, we focused on the inhibitory effects of REs on α-Glu activity and the types of inhibition, and elucidated the mechanism of interaction between REs and α-Glu. We found that ultrasonic treatment promoted the accumulation of rose polyphenols, which increased the inhibitory effect of REs on α-Glu activity. REs were hybrid inhibitors of α-Glu and the fluorescence quenching of α-Glu indicated that REs could statically quench the endogenous fluorescence of α-Glu and form a noncovalent complex with α-Glu mainly through hydrogen bonding. Fluorescence spectroscopy and CD analysis showed that REs increased the chance of polypeptide chain misfolding and disrupted the natural conformation of the enzyme by altering the microenvironment around the tryptophan and tyrosine residues, ultimately leading to a decrease in the activity of α-Glu. Molecular docking simulation experiments further verified the above conclusion that, in comparison, polyhydroxy phenolics had a higher fit to the active site of α-Glu, explaining the stronger inhibition of α-Glu by REs with high polymerization and numerous phenolic hydroxyl groups. In conclusion, REs showed potential as natural alternatives to α-Glu inhibitors, providing a rationale for their use in food and medicine.

## Figures and Tables

**Table 1:** Total phenolic content and α-glucosidase inhibitory effect of rose extracts (REs).

Samples	REs (Control)	REs (UP)	Acarbose
Total phenolic content (mg/g)	134.91 ± 5.69 ^a^	167.40 ± 2.67 ^b^	/
IC_50_ α-Glu (μg/mL)	1.96 ± 0.07 ^A,B^	1.33 ± 0.10 ^C^	0.17 ± 0.01

**Note:** UP denotes ultrasound pretreatment; ^a,b^ denotes a significant difference between the total phenolic content of REs (Control) and REs (UP) (*p* < 0.05); ^A^ denotes a significant difference between the IC_50_ of ERs (Control) and ERs (UP) (*p* < 0.05); ^B^ denotes a significant difference between the IC_50_ of ERs (Control) and acarbose (*p* < 0.05); ^C^ denotes a significant difference between the IC_50_ of ERs (UP) and acarbose (*p* < 0.05).

**Table 2:** Effect of REs on fluorescence quenching parameters and thermodynamic parameters of α-Glu.

T (K)	*K_sv_* (L/g)	*Ka* (L/g)	*n*	*ΔH°* (kJ/g)	*ΔG°* (kJ/g)	*ΔS°* (J/g/K)
298 K	1457.11	3019.95	1.11	−35.04	−20.05	−50.31
304 K	1271.63	2884.03	1.12		−19.75	
310 K	1147.02	1737.80	1.06		−19.44	

**Table 3:** Docking scores of the main phenolic compounds with critical amino acid residues surrounding the active pocket of α-Glu.

Chemical Compound	Affinities (kcal/mol)	Active Amino Acid Residues
Acarbose	−8.70	Tyr158, Asn415, Asp307, Ser304, Ser157, Thr310, Arg315, Glu411, Phe159, Tyr316, Gly309, Phe303, Ser311, Pro312, Val308, Ala329, Phe314, Gly160, Leu313, Lys156, Ser240, Asp242
Catechin	−8.60	Glu277, Asp307, Asp352, Tyr158, Arg315, Arg442, Gln279, His280, Phe303, Arg213, Phe178, Phe314, Phe159, Glu411
Quercetin-3β-D-glucoside	−9.60	Pro312, Lfu246, His280, Ser311, Gin353, Asp307, Thr310, Phe303, Asp242, Glu277, Gln279, Arg315, Ser240, Arg442, Glu411, Tyr158, Lys156
Proanthocyanidin B2	−9.90	Lys156, Ser240, Tyr158, Phe314, Pro312, Phe303, Thr3306, Arg315, Ser311, Gln353, Thr310, Asp307, His280, Asp242, Gln279, Asp352, Leu246
Gallic acid	−6.50	Leu313, Asn317, Phe314, Asn235, Lys156, Phe433, Glu429, ILE419, His423, Glu422, Ser236, Thr237, Ala418
Rutin	−10.10	Asp352, Arg442, Asp242, Phe178, Gln279, Ser40, Phe303, Phe159, Glu411, Pro312, Tyr316, Tyr158, Leu313, Val232, Asn415, Phe314, Ser311, Asp233, Thr310, His280, Arg315, Asp307, Lys156, Ser157

## Data Availability

The original contributions presented in the study are included in the article, further inquiries can be directed to the corresponding authors.
